# Effect of Nanomaterials on Gut Microbiota

**DOI:** 10.3390/toxics11040384

**Published:** 2023-04-17

**Authors:** Ying Ma, Jiahe Zhang, Nairui Yu, Jiaqi Shi, Yi Zhang, Zhangjian Chen, Guang Jia

**Affiliations:** 1 Department of Occupational and Environmental Health Sciences, School of Public Health, Peking University, Beijing 100191, China; 2 Beijing Key Laboratory of Toxicological Research and Risk Assessment for Food Safety, School of Public Health, Peking University, Beijing 100191, China

**Keywords:** nanomaterials, gut microbiota, antimicrobial properties, oxidative stress, titanium dioxide nanoparticles

## Abstract

Nanomaterials are widely employed in everyday life, including food and engineering. Food additives on a nanoscale can enter the body via the digestive tract. The human gut microbiota is a dynamically balanced ecosystem composed of a multitude of microorganisms that play a crucial role in maintaining the proper physiological function of the digestive tract and the body’s endocrine coordination. While the antibacterial capabilities of nanomaterials have received much interest in recent years, their impacts on gut microbiota ought to be cautioned about and explored. Nanomaterials exhibit good antibacterial capabilities in vitro. Animal studies have revealed that oral exposure to nanomaterials inhibits probiotic reproduction, stimulates the inflammatory response of the gut immune system, increases opportunistic infections, and changes the composition and structure of the gut microbiota. This article provides an overview of the impacts of nanomaterials, particularly titanium dioxide nanoparticles (TiO_2_ NPs), on the gut microbiota. It advances nanomaterial safety research and offers a scientific foundation for the prevention, control, and treatment of illnesses associated with gut microbiota abnormalities.

## 1. Exposure of the Gut Microbiota to Nanomaterials

Nanomaterials (NMs) are materials with unique properties that are made up of nanostructured basic units or at least one dimension at the nanoscale (geometric scales ranging from 1 nm to 100 nm), such as nanopowders, nanofibers, nanofilms, nanoblocks, and nanopores. According to the classification of chemical composition, they can be divided into metal nanomaterials, nanocrystalline materials, inorganic nonmetallic materials, polymer nanomaterials, and nanocomposites. Nanomaterials exhibit characteristics of a small size effect, high specific surface area, and quantum size effect [1]. Nanomaterials have a wide range of uses due to their superior physical and chemical characteristics, such as biomedicine, diagnostic imaging, DNA nanotechnology, biosensing, and drug-loaded treatment [2]. Notably, nanomaterials have several uses in food engineering [3]. They can be utilized as coatings to minimize mechanical damage or microbiological contamination and improve food color and flavor. Nanocapsules can be employed as carriers to enter, protect, and transport active chemicals in food and medications while preserving the product’s appearance and taste [4]. Nanofilms are commonly utilized in chocolate, confectionery, baked goods, and other food-related products because they protect food surfaces from moisture, oil, and gas [5]. Currently, whether it is food itself, food packaging, or the entire process of food manufacturing and production, using different nanomaterials is unavoidable, which undoubtedly increases people’s intestinal exposure risk.

Despite their importance in medicine, engineering, food processing, and other fields, the safety of nanomaterials remains a major concern. One of the current and future focuses of nanomaterials is the study of their biological effects and toxicity. To date, many in vivo and in vitro studies have been conducted on a range of nanomaterials, such as nano-TiO_2_, SiO_2_, carbon nanotubes, fullerenes, and iron nanoparticles, demonstrating their impact on redox balance and metabolism. Many safety assessments of oral exposure to nanomaterials have revealed that they harm the human digestive system. Therefore, the purpose of this review is to investigate the effects of nanomaterials represented by titanium dioxide nanoparticles on the gut microbiota and to propose ideas for nanomaterial safety evaluation.

## 2. The Function of the Gut Microbiota

With 10^14^–10^15^ microorganisms in the gut, such a high population plays an important part in human health [6]. The primary function of the gut microbiota is to process undigested foods such as protein and dietary fiber [7]. The gut microbiota contains a variety of enzymes that aid in carbohydrate digestion, including glycoside hydrolases, glycosyltransferases, glycosyltransferases, and carbohydrate esterases [8]. The gut microbiota creates short-chain fatty acids (SCFAs) through the anaerobic fermentation of carbs, the majority of which are made up of acetic acid, propionic acid, isobutyric acid, butyric acid, isovaleric acid, and valeric acid. Short-chain fatty acids facilitate contact between the intestinal microbiota and the host, as well as the regulation of cell growth and differentiation [9,10,11]. For example, butyrate, which is the most abundant in production, at physiological concentrations promotes cell differentiation and inhibits growth [12,13]. Butyrate functions as an agonist of histone deacetylase (HDCA) inhibitors and histone transferases, boosting histone acetylation and promoting post-translational histone modification [14,15]. Histone deacetylase inhibitors prevent cell growth by halting the cell cycle [16]. Butyrate triggers Caco-2 cell differentiation and alkaline phosphatase activation, as well as cell interleukin 8 (IL-8) release [17].

The gut microbiota can also influence host immunity. When compared to normal mice, germ-free (GF) mice had undeveloped immune systems, as proven by lower antimicrobial peptide expression, lower IgA production, fewer T-cell types, and higher microbial sensitivity [18]. In normal mice treated with antibiotics, Clostridiales decreased, causing a drop in T regulatory (Treg) lymphocytes in the gut [19]. Tregs are the primary regulators of immune tolerance and inflammation as T cells that can suppress the proliferation of Th0 cells. Treg dysregulation is often closely linked to intestinal autoimmunity, such as causing inflammatory bowel disease (IBD) when Treg anti-inflammatory activity is decreased [20]. Moreover, the gut microbiota encourages the proliferation of the CD4 T-cell population [21], which is the primary source of IL-22 in the gut and is important in the regulation of intestinal inflammation [22]. The immunomodulatory protein polysaccharide A (PSA) from *Bacteroides fragilis* promotes the conversion of CD4 (+) T cells to Foxp3 (+) Treg cells [23], promoting the establishment of immune tolerance [24].

The gut microbiota is also a key regulator of host metabolism, influencing host energy balance, glucose metabolism, and lipid metabolism [25]. The gut microbiota can react with the fatty acid duplex in food to form metabolites that the host cannot synthesize, such as conjugated linoleic acid (CLA). Conjugated linoleic acid reduces insulin sensitivity and atherosclerosis by inhibiting the expression of PPARγ and LXRα [26,27,28]. The fatty acids generated by lactic acid bacteria in the gut drive adipocyte differentiation by activating PPARγ, as well as boosting adiponectin synthesis and glucose absorption, which influences glycolipid metabolism [29]. When compared to GF mice, normal mice had greater metabolic levels of pyruvate, citric acid, fumaric acid, and malic acid while having lower blood triglyceride levels, altering host energy and lipid metabolism [30].

## 3. Antimicrobial Properties of Nanomaterials

Most nanomaterials have antibacterial properties that are effective against common bacteria. Metal oxide nanomaterials such as nano-TiO_2_, ZnO, and Ag_2_O can inhibit common bacteria such as *E. coli*, *Bacillus subtilis*, and *Staphylococcus aureus* [31]. Nano-TiO_2_ and ZnO are poisonous to gram-negative, gram-positive, and fungal microorganisms [32]. Even in the absence of UV irradiation, nano-TiO_2_ retains its antibacterial ability against *E. coli* [33]. The antibacterial ability of nanomaterials is affected by their size, production process, and crystal form. Moreover, the temperature, pH, and ionic strength of the environment also have an impact on the antibacterial capabilities of nanomaterials. Smaller particle size nano-TiO_2_ and the anatase phase have been shown to be more harmful to *E. coli*; nevertheless, the toxicity of nano-TiO_2_ diminishes with increasing pH (5.0–10.0) and ion concentration [34]. It is worth noting, however, that the above characteristics do not apply to fungi associated with plant rhizomes. According to reports, there were no effects of nanomaterial type, concentration, or charge on the community structure of either rhizobia or AM fungi colonizing plant roots [35].

**Figure 1:**
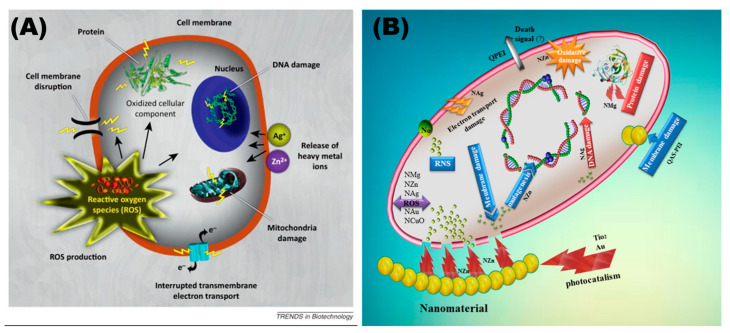
The provision of various antibacterial mechanisms by nanoparticles. Nanoparticles and the ions they release produce free radicals that induce oxidative stress, which induces bacterial death. (**A**) Reproduced with permission [31]. Copyright 2012, Elsevier. (**B**) “?” represented that there is no consensus on the signal of bacterial death caused by nanomaterials. Reproduced with permission [41]. Copyright 2017, Elsevier.

There are several possible hypotheses for the antibacterial mechanism of nanomaterials (Figure 1). The electronegative complex groups on the bacterial membrane can attract each other with electropositive metal ions, causing metal nanomaterials to accumulate on the bacterial surface and enter the cell, altering the permeability of the bacterial membrane and allowing bacterial contents to leak out [36]. Nanomaterials that enter bacteria can also alter the function of enzymes and proteins, interfering with the bacterium’s regular physiological metabolism [37]. Antimicrobial properties in nanomaterials can also be produced through oxidative stress [38]. H^+^ dispersed on the surface of metal nanoparticles can oxidize OH^−^ and H_2_O to OH. As a powerful oxidant, ·OH causes bacterial redox imbalance. Under UV irradiation, this behavior will be more severe [39]. Nanomaterials offer a unique multiple antibacterial mechanism and have a good killing impact on a range of drug-resistant bacteria when compared to typical disinfectants and medicines [40]. As a result, nanomaterials may offer a solution to multidrug-resistant bacteria.

## 4. Effects of Nanomaterials on Gut Microbiota

### 4.1. Titanium Dioxide Nanoparticles (TiO_2_ NPs)

**Figure 2:**
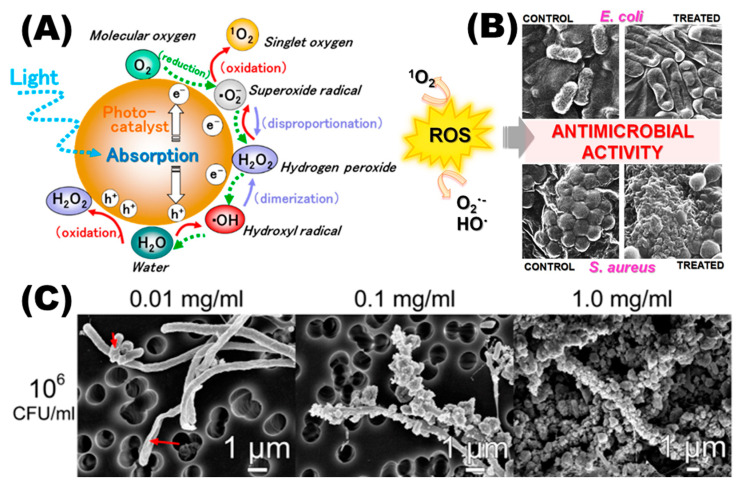
Titanium dioxide causes oxidative stress, which has an antimicrobial effect. (**A**) TiO_2_ NPs generate reactive oxygen during the photocatalytic reduction and oxidation of oxygen and water. Reproduced with permission [52]. Copyright 2017, American Chemical Society. (**B**) Damage of TiO_2_ NPs to *E. coli* and *S. aureus*. Reproduced with permission [53]. Copyright 2019, MDPI. (**C**) Scanning electron microscopy images of *E. coli* bacterial cells exposed to TiO_2_ NPs at various concentrations (0.01, 0.1, and 1.0 mg/mL). The initial bacterial concentration was 10^6^. Reproduced with permission [54]. Copyright 2016, *Nature*.

TiO_2_ NPs have limited impacts on gut microbiota, as evidenced by acute or subchronic experiments that have limited influence on gut microbiota diversity but have a greater impact on gut microbiota quantity (Figure 2, Table 1). Among these, TiO_2_ NPs have a significant impact on bacteria, particularly *Lactobacillus*, *Firmicutes*, and *Proteobacteria* [33,34,35].

Subacute or subchronic exposure to TiO_2_ NPs had less of an effect on the gut microbiota in typical rodent models. Li et al. [42] treated mice with TiO_2_ NPs (100 mg/kg) for 28 days and observed that TiO_2_ NPs did not affect the diversity of gut microbiota but modified the composition structure of the microbiota, in which the abundance of *Proteus* was reduced dramatically. Wei et al. [43] investigated the long-term toxicity of TiO_2_ NP exposure. Weaned young mice were given TiO_2_ NPs for three months, and their body weight was found to be lower than that of the control group, which intensified the chronic colitis and immunological response generated by dextran sulfate sodium salt (DSS). According to research, TiO_2_ NPs have no effect on the diversity of gut microbiota but drastically affect the quantity of probiotics such as *Bifidobacteria* and *Lactobacilli*. Chen et al. [44] found that after 30 days of oral treatment (2, 10, and 50 mg/kg) the structure and composition of the rat gut microbiota were altered, resulting in significant increases in *L. gasseri* and *Turicibacter*, while *Veillonella* was dramatically reduced in the exposure group at 14 days. After 28 days, the abundance of *L. gasseri* continued to increase significantly, as did *L.NK4A136_group*. Another study demonstrated that TiO_2_ NPs (2, 10, and 50 mg/kg) significantly enhanced the abundance of *Lactobacillus* and *Allobaculum* and decreased the abundance of *Adlercreutzia* and *unclassified Clostridiaceae* in the exposed group following 21 days of subchronic exposure [45]. In the population, the average long-term intake of titanium dioxide is 0.06 mg/kg bw/day for people over 70 years old, 0.17 mg/kg bw/day for people aged 7–69 years, and 0.67 mg/kg bw/day for children aged 2–6 years [46]. The dose settings of the above animal experiments were considered with a safety factor (100×), which well reflects the situation after TiO_2_ NPs exposure.

TiO_2_ NPs also have an impact on the gut microbiota in other animal or in vitro models. When TiO_2_ NPs were coexposed to bisphenol A (BPA), they increased the abundance of *Lawsonia* in *Danio rerio* while decreasing the abundance of *Hyphomicrobium* [47]. Dudefoi et al. [48] used food-grade TiO_2_ NPs to imitate human digestive system dosages in an in vitro model. Following two days of bacterial culture, there were very minor impacts on the gut microbiota. *Clostridium cocleatum* increased in abundance, whereas *Bacteroides ovatus* decreased. In vitro studies show that TiO_2_ NPs can still have antibacterial properties. According to Albukhaty et al. [49], TiO_2_ NPs can effectively inhibit *Staphylococcus aureus* and *Escherichia coli* activity in vitro.

TiO_2_ NPs can disrupt the tight junctions of intestinal epithelial cells, producing a loss of intestinal barrier structure and altering the diversity and composition of gut microbiota communities in organisms. Li et al. [42] examined the two primary TiO_2_ NPs crystals, anatase and rutile, and discovered that the latter had a greater influence on the intestinal ecological habitat of mice. Long intestinal villi and an uneven arrangement of villus epithelial cells were observed in mice fed rutile. However, in the Chen experiment, the intestinal shape of rats was changed significantly by anatase, as evidenced by inflammatory infiltration and mitochondrial abnormalities [44]. Obese mice were more susceptible to this. Mice fed a high-fat diet and exposed to TiO_2_ NPs experienced goblet cell loss, the structural distortion of crypts, and the infiltration of inflammatory cells around crypts. The number of dendritic cells and macrophages in the colonic mucosa increased significantly, as did the levels of IL-12, IL-17, KC/GRO, and IL-10 [50].

Moreover, TiO_2_ NPs may be hazardous to other digestive system organs, which might have an indirect impact on the gut microbiota. Li et al. [42] showed that TiO_2_ NPs accumulated in the spleen, lungs, and kidneys affected the shape and organization of intestinal epithelial cells and altered the composition of gut microbiota over time. Chen et al. [51] discovered that the gut–liver axis regulating mechanism may play a significant role in the influence of nanomaterials on gut microbiota. In rats, subchronic oral TiO_2_ NP treatment produces hepatotoxicity, including hepatocyte steatosis and mitochondrial dysfunction. Substantial changes in the alanine, aspartate, and glutamate pathways and metabolic pathways may be critical metabolic pathways leading to disruptions in energy metabolism and oxidation/antioxidant imbalances. A significant increase in the synthesis of lipopolysaccharide (LPS) by the gut microbiota in rats might be proof of the connection between liver metabolism disorder and gut microbiota dysregulation. 

**Table 1:** Effects of TiO_2_ NPs on gut microbiota.

Animal	Physicochemical Properties	Exposure Dose	Exposure Time	Antibacterial Activity	Others
Albino mice [55]	Hexagonal (25.12 nm)	50 μg, 100 μg	18 d	*Firmicutes* ⬇	
C57BL/6 [45]	Spherical E171 (28–1158 nm)	2, 10, 50 mg/kg	21 d	*Levilactobacillus* ⬆ *Allobaculum* ⬆ *Adlercreutzia* ⬇ Unclassified *Clostridiaceae* ⬇	
C57BL/6 [56]	Anatase (25 nm)	1 mg/kg	7 d	*Bifidobacterium* ⬇	
C57BL/6 [42]	Rutile	100 mg/kg	28 d	*Proteobacteria* ⬇	The small intestine villi were long, and the villi epithelial cells were arranged irregularly.
C57BL/6J [43]	Anatase (10 nm, 50 nm)	diets containing 0.1% TiO_2_ NPs	90 d	*Bifidobacterium* ⬇ *Lactobacillus* ⬇	The body weight was lower than that of the control group, and it exacerbated the chronic colitis and immune response induced by Dextran Sulfate Sodium Salt (DSS).
Sprague–Dawley rats [44]	Anatase	2, 10, 50 mg/kg	28 d	*L. gasseri* ⬆ *L.NK4A136_group* ⬆	Pathological inflammatory infiltrates and mitochondrial abnormalities cause significant alterations in the shape of the gut.
Sprague–Dawley rats [57]	Anatase (25.2 nm)	100 mg/kg	14 d	*Anaerobium* ⬆ *Prevotella* ⬆ *Granulicatella* ⬆ *Lactobacillaceae* ⬇	

### 4.2. Silver Nanoparticles (Ag NPs)

**Figure 3:**
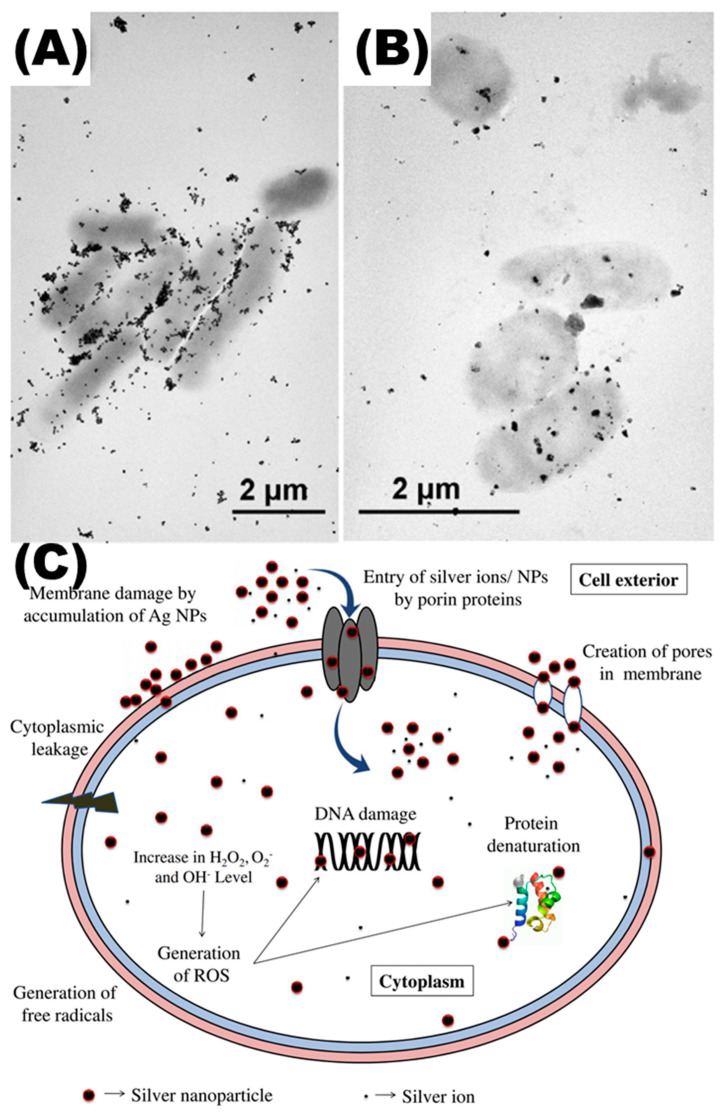
Effects of Ag NPs on gut microbiota. (**A**,**B**) The TEM images of *E. coli* co-incubated with Ag NPs. Reproduced with permission [64]. Copyright 2015, American Chemical Society. (**C**) Mechanisms of Ag NPs’ impact on bacterial cells. Reproduced with permission [65]. Copyright 2018, Elsevier.

Silver NPs are one of the most extensively researched antimicrobial noble metal nanoparticles, with strong antibacterial activity against a wide range of diseases, including drug-resistant bacteria (Figure 3, Table 2). Silver NPs can alter the diversity and composition of gut microbiota, and the effect is relatively consistent across species. Specifically, this increases the amount of gram-negative bacteria in the gut microbiota, primarily affecting *Lactobacillus* of the *Firmicutes* phylum and *E. coli* of the *Proteobacteria* phylum [58]. Han et al. [59] discovered that the gut microbiota diversity of fruit flies was dramatically reduced after Ag NP exposure, with the abundance of *Acetobacter* dropping while *Levilactobacillus brevis* had a stronger advantage.

In vitro, Ag NPs have strong antibacterial capabilities, and the mechanism is assumed to be direct contact and oxidative stress. The former considers that Ag NPs can slowly release silver ions and covalently bind to sulfhydryl groups (-SH) in proteins, rendering them inactive [60]; the latter believes that Ag NPs catalyze the synthesis of huge amounts of reactive oxygen species (ROS) from water and oxygen, damaging cellular genetic material and triggering apoptosis. Studies have revealed that both pathways occur, with oxidative stress being the primary mechanism of Ag NP antibacterial activity, while silver ions have a limited impact [61,62].

In vivo, the antibacterial mechanism is connected to immunological regulation. Williams et al. [58] investigated the effects of different sizes of nanosilver and silver acetate on gut microbiota and mucosal gene expression in SD rats. Low dosages and small sizes of Ag NPs were discovered to change intestinal gene expression, resulting in the reduced expression of critical immunomodulatory genes such as MUC3, TLR2, TLR4, GPR43, and FOXP3.

Oral Ag NPs affect animal growth and development, but their advantages and risks remain unknown. Fondecila et al. [63] discovered that Ag NPs may decrease the abundance of *E. coli* linearly in vitro. When giving Ag NPs to piglets, their daily feed intake and weight rose linearly with the dosage of Ag NPs. At the same time, the concentration of *E. coli* in feces was reduced, whereas the concentration of *Lactobacilli* was unaffected. Silver NPs altered the composition of the piglet gut microbiota, which benefits development and metabolism. Han et al. [59] discovered that the toxicity of Ag NPs was greater than that of microsilver in fruit flies. Although Ag NPs have no effect on adult fruit flies, they do reduce the rate of development and reproduction. In conclusion, the interference of Ag NPs in gut microbiota may be due to their own antibacterial properties, and an imbalanced gut microbiota exacerbates Ag NP toxicity.

**Table 2:** Effects of Ag NPs on gut microbiota.

Animal	Physicochemical Properties	Exposure Dose	Exposure Time	Antibacterial Activity	Others
C57BL/6 [66]	22.2 ± 6.1 nm	0.1, 2, 40 μg	120 d	*Firmicutes* ⬆ *Bacteroidetes* ⬇	Changes in liver metabolism
C57BL/6 [67]	55.17 ± 2.67 nm	46, 460, 4600 μg/kg	28 d	*Firmicutes* ⬆ *Bacteroidetes* ⬇	
C57BL/6J [68]	60–150 nm	0.5, 2.5 mg/kg	14 d 28 d	*Lachnospiraceae* ⬆ *Bacteroidetes* S24-7 ⬇	Accumulates in the liver, spleen, and lungs.
Wistar rats [69]	7 nm	100 mg/kg	28 d	*Bacteroidota* ⬆ *Verrucomicrobia* ⬇ *Proteobacteria* ⬇ *Lactobacillaceae* ⬇	Minor inflammatory cell infiltration in the submucosa of the gastric mucosa; there are small yellowish to dark granules in the submucosa and macrophages at the tip of the duodenal villi.
Sprague–Dawley rats [70]	Spherical (50 nm) cube (45 nm)	3.6 mg/kg	14 d	Cube: *Clostridium* spp. ⬇ *Bacteroides uniformis* ⬇ *Christensenellaceae* ⬇ *Coprococcus eutactus* ⬇ Spherical: *Coprococcus eutactus* ⬇ *Dehalobacterium* spp. ⬇ Peptococcaeceae ⬇ *Corynebacterium* spp. ⬇ *Aggregatibacter pneumotropica* ⬇	
Sprague–Dawley rats [58]	10, 75, 110 nm	18, 36 mg/kg	91 d	*Bifidobacterium* ⬆ *Firmicutes* ⬇	The expression level of MUC3, TLR2, TLR4, GPR43, FOXP3 were decreased.
Broiler chickens [71]	50 nm	25, 50, 75 ppm	42 d	Total anaerobic bacteria ⬇ *Escherichia coli* ⬇	It had side effects on the immune mechanism.
Zebrafish [72]		10, 33, 100 μg/L	45 d	*Proteobacteria* ⬆	
*Drosophila* melanogaster [59]	7 μm 1.5 μm	450 mg/mL	7 d	*Acetobacter* ⬇	
Weaned pigs [63]		20, 40 mg/kg	14 d	*Coliforms* ⬇	

### 4.3. Zinc Oxide Nanoparticles (ZnO NPs)

ZnO NPs have a strong antibacterial effect and inhibit a wide range of bacteria in the gut (Table 3). They have the potential to alter the diversity and composition of the gut microbiota; for example, the abundance of gut probiotics such as *Lactobacillus* was increased. After 28 days of ZnO NP 1000 mg/kg administration to rats, the abundance of several *Lactobacillus* probiotics in the intestines of female rats increased significantly [73].

**Figure 4:**
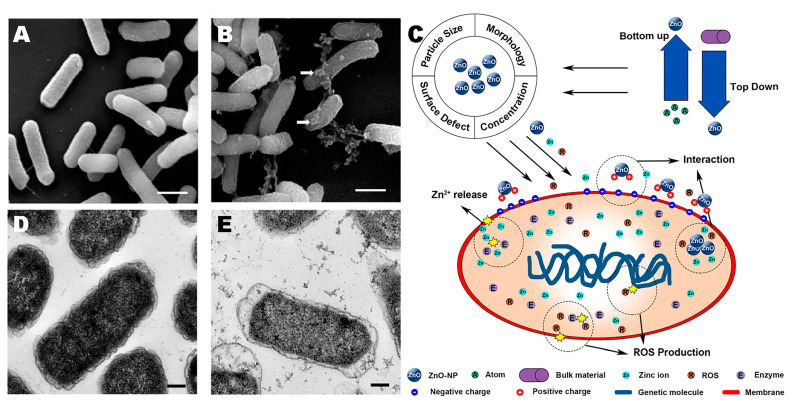
Effects of ZnO NPs on gut microbiota. The SEM (**A**,**B**) and TEM (**D**,**E**) images of *E. coli* treated without ZnO NPs (**A**,**D**) and 20 mM ZnO NPs (**B**,**E**). White arrows indicate ZnO NPs. Scanning electron microscopy scale bar = 1 μm, TEM scale bar = 0.2 μm. Reproduced with permission [79]. Copyright 2021, MDPI. (**C**) ZnO NP antibacterial mechanism and influencing factors schematic diagram. Reproduced with permission [80]. Copyright 2020, Dovepress.

There are several hypotheses about the antibacterial mechanism of ZnO NPs (Figure 4). Antimicrobial processes such as oxidative stress, direct interaction with bacteria, and zinc ion release are all considered feasible. Several investigations have suggested that oxidative stress is the primary antibacterial mechanism of ZnO NPs. In an aqueous solution, ZnO NPs may generate •OH, singlet oxygen or superoxide anions (O_2_•^−^), and hydrogen peroxide (H_2_O_2_). The larger the ZnO NP surface area is, the higher the ROS output [74,75]. During the direct interaction of ZnO NPs with *E. coli*, the ROS generated trigger the oxidation of the lipid membrane in the cell wall, leading to the leakage of cell contents [76]. Zinc ions produced by ZnO NPs are considered to have antibacterial properties. Nevertheless, ZnO NPs have limited solubility and are sensitive to ambient pH. ZnO NPs tend to remain intact at neutral pH, but in acidic conditions ZnO NPs dissolve and release zinc ions that bind to biomolecules (proteins, carbohydrates, etc.) in bacteria and impede their development [77,78].

**Table 3:** Effects of ZnO NPs on gut microbiota.

Animal	Physicochemical Properties	Exposure Dose	Exposure Time	Antibacterial Activity	Others
Weaned piglets [81]	23 nm	diets containing 0.3, 0.4, 0.5, 0.6 g/kg ZnO NPs	14 d	*Lactobacillaceae* ⬆ *Coliforms* ⬇	Improves growth performance, reduces the incidence of diarrhea, regulates immune status and antioxidant activity.
Weaned pigs [82]	71.61 nm	150, 300, 450, 3000 mg/kg	21 d	*Coliforms* ⬇	Reduces diarrhea and improves intestinal morphology.
Weaned piglets [83]	23 nm	600 mg/kg	14 d	Ileum: *Proteobacteria* ⬆ *Firmicutes* ⬇ Cecum: *Firmicutes* ⬆ Colon: *Firmicutes* ⬆ *Bacteroidetes* ⬇	Reduces diarrhea and improves intestinal morphology.
Wistar albino rats [73]		1000 mg/kg	28 d	Male: *Firmicutes* ⬆ *Bacteroidetes* ⬇ Female: *Firmicutes* ⬇ *Verrucomicrobia* ⬆	
C57BL/6 [84]	50 nm	26 mg/kg	30 d	*Actinobacteria* ⬇	
Hens [85]	30 nm	25, 50, 100 mg/kg	63 d	SMB53 ⬆ *Proteus* ⬇ *Lactobacillus* ⬇	
Cyprinus carpio [86]		diets containing 500 mg/kg ZnO NPs	42 d	*Flavobacteriumspecies* ⬆ *Aeromonasspp* ⬆	

### 4.4. Carbon-Based Nanomaterials (CNMs)

**Figure 5:**
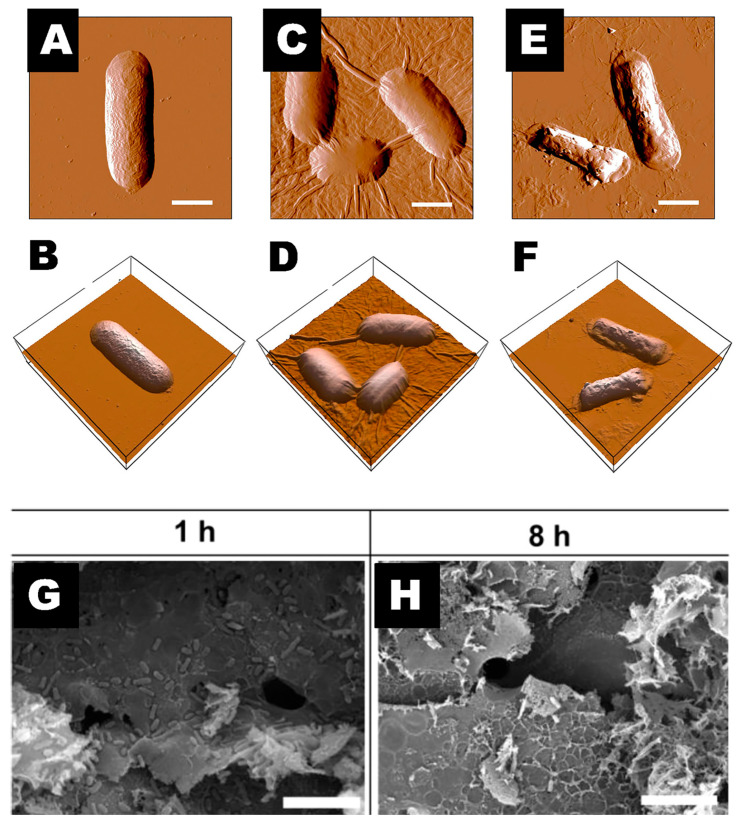
Atomic force microscopy and 3D images of *E. coli* cells after incubation with graphene oxide (GO). Incubate *E. coli* with deionized water (**A**,**B**), 40 μg/mL GO-0 (**C**,**D**) and 40 μg/mL GO-240 (**E**,**F**) for 2 h. Scale bar is 1 μm. Reproduced with permission [94]. Copyright 2012, American Chemical Society. (**G**,**H**) Scanning electron microscopy images of *E. coli* incubated with laser-induced graphene (LIG) 1 and 8 h. Scale bar is 10 μm. Reproduced with permission [95]. Copyright 2020, American Chemical Society.

Carbon-based nanomaterials with at least one dimension less than 100 nm. There are several common types, such as fullerenes, carbon nanotubes (CNTs), carbon dots, and graphene and its derivatives. Carbon-based nanomaterials can exert antibacterial properties via a variety of mechanisms, including physical destruction, the inflammatory immune response, and oxidative stress (Figure 5, Table 4).

One of the most typical antimicrobial mechanisms in CNMs is the physical destruction of the outer cell membrane or cell wall. Carbon-based nanomaterials bind to peptidoglycan and proteins in the cell membrane, causing cell membrane rupture [87,88]. Carbon nanotubes and graphene, for example, have sharp edges that may puncture bacterial membranes, resulting in the release of bacterial internal components such as RNA [89,90].

The metabolic inflammatory response is linked to changes in the gut microbiota caused by CNMs. Carbon nanotubes boosted the release of inflammatory factors such as IL-1β, IL-6, and TNF-α in the duodenum and colon, as well as the transition of the phylum *Firmicutes* to *Bacteroidetes* and the abundance of the pro-inflammatory bacteria *Alitipes_uncultured* and *Lachnospiraceae bacterium* A4 [91].

Oxidative stress is another major antibacterial mechanism in CNMs. Carbon quantum dots generate ROS when exposed to blue light, dramatically inhibiting the activity of methicillin-resistant *Staphylococcus aureus* and *Escherichia coli* [92]. Graphene oxide and reduced graphene oxide also showed dose-dependent antibacterial action against *Pseudomonas aeruginosa* by creating ROS, with graphene oxide inducing bacterial DNA fragmentation [93].

**Table 4:** Effects of CNMs on gut microbiota.

Animal	Physicochemical Properties	Exposure Dose	Exposure Time	Antibacterial Activity	Others
CD-1 (ICR) mice [91]	SWCNT diameter: 1.04–1.17 nm, length: 1–5 μm	0.05, 0.5, 2.5 mg/kg	7 d	*Bacteroidetes* ⬆ *Lachnospiraceae bacterium* A4 ⬆	Histological lesion scores increased, intestinal permeability increased, and the levels of pro-inflammatory cytokine (IL-1β, IL-6, and TNF-α) increased.
C57BL/6 [96]	MWCNT diameter: 10.7 ± 3.1 nm	2.8 mg/kg	28 d	*Firmicutes* ⬆ *Tenericutes* ⬆ *Bacteroidetes* ⬇ *Proteobacteria* ⬇	Induced inflammation of the lungs.
C57BL/6 [97]	MWCNT diameter: 20–30 nm, length: 0.5–2 μm	5 μg/kg	15 d	*Verrucomicrobia* ⬆ *Bacteroidetes* ⬇	

### 4.5. Effects of Other Nanomaterials on Gut Microbiota

Silica nanoparticles (SiO_2_ NPs) can affect the abundance of gut microbiota through inflammatory immune responses (Table 5). Following 7 days of administering 2.5 mg/kg bw/day SiO_2_ NPs to mice, pro-inflammatory factors such as IL-1β, IL-6, and TNF-α increased considerably in the small intestine and colon. Meanwhile, the phylotypes responded to the *Firmicutes* increase (39.9% vs. 26.1% in control mice) and *Bacteroidete* decline [98].

Copper-loaded chitosan nanoparticles (CNP-Cu) can increase the abundance of *Bifidobacterium* and *Lactobacillus* since some microbiota were inhibited by CNP-Cu [99]. Wang et al. fed weaned piglets CNP-Cu to investigate its effects. The results showed that the abundance of *E. coli* was dramatically reduced but the numbers of *Lactobacillus* and *Bifidobacterium* were increased [100].

In vitro investigations revealed that the richness of the microbiota increased dose-dependently when exposed to nano-Al_2_O_3_. The structure of the gut microbiota was altered dramatically at high dosages (50 mg/L), with the number of *Firmicutes* and *Proteobacteria* increasing and *Bacteroidetes* decreasing [101].

**Table 5:** Effects of other nanomaterials on gut microbiota.

Animal	Nanomaterials	Physicochemical Properties	Exposure Dose	Exposure Time	Antibacterial Activity	Others
Weaned piglets [100]	Copper-loaded chitosan nanoparticles (CNP-Cu)	diameter: 121.9 nm, width: 23.1 nm	100 mg/kg	28 d	*Levilactobacillus* ⬆ *Bifidobacterium* ⬆ *Escherichia coli* ⬇	The piglets’ average daily weight increased, feed intake increased, and the rate of diarrhea decreased; increased length of intestinal epithelial villi.
Broiler chickens [102]	nanoselenium		0.075, 0.15, 0.3 mg/kg	42 d	*Lactobacilli* ⬆ *Coliforms* ⬇	Improves intestinal morphology and immune function.
CD-1 (ICR) mice [98]	SiO_2_ NPs	10.8 ± 1.7 nm	2.5 mg/kg	7 d	*Firmicutes* ⬆ *Proteobacteria* ⬆ *Bacteroidetes* ⬇ *Lactobacillus* ⬇	Increased pro-inflammatory cytokines in the intestine.
Broiler chickens [103]	Iron nanoparticles	50 ± 15 nm	8 mg/kg	42 d	*Lachnospiraceae* ⬆ *Bacteroidaceae* ⬆, *Alistipes* ⬆ *Rikenellaceae* ⬆ *Lactobacillaceae* ⬇ *Anaerobes* ⬇	
	Copper nanoparticles	55 ± 15 nm	1.7 mg/kg	42 d	*Rumen_occoccidae* ⬆ *genus Blautia* ⬆ *Bacteroides* ⬆ *Firmicutes* ⬇ *Lactobacillaceae* ⬇ *Rikenellaceae* ⬇	
	A mixture of Cu and Zn asparaginates	65 ± 15 nm	2.84 mg/kg	42 d	*Rumen occoccidae* ⬆ *Bacteroides* ⬆ *Firmicutes* ⬇ *Lactobacillaceae* ⬇ *Rikenellaceae* ⬇	

## 5. Summary and Future Outlooks

The structure and abundance of the gut microbiota are dynamic and influenced by dietary properties. According to the accessible data, *Firmicutes* were discovered to be the most susceptible microbiota. *Firmicutes* are one of the most numerous bacterial families in the gut. *Lactobacilli*, which function as a probiotic in *Firmicutes*, were sensitive to nanoparticles. Another probiotic called *Bifidobacterium* was another sensitive microbiota, with increased abundance when exposed to Ag NPs and CNP-Cu NPs and reduced abundance when exposed to titanium dioxide. It can be seen that the change of microbiota is material-specific.

Over the past few decades, rapid advancements in nanomaterials have provided intriguing alternatives to antibacterial therapies. Nanomaterials, as opposed to regular antibiotics, are less prone to causing bacterial resistance. They alter the structure of the gut microbiota, influencing host health by triggering the intestinal immune system and oxidative stress. Unfortunately, most research on the impact of nanomaterials on gut microbiota is restricted to animal or in vitro tests, and studying complicated human environments remains difficult. Since there are still few data on the real exposure concentrations of nanomaterials, dose selection in animal studies needs to be carefully considered. In future, experiments should focus on the influence of nanomaterials on the human gut microbiota, bridging the gap between microbiota disorders and host illnesses and supplementing the safe use of nanomaterials.

## Data Availability

Not applicable.
